# Daily Quiz-Based Microlearning Program to Support Electrocardiogram Interpretation Training for Medical Students: A Feasibility Study

**DOI:** 10.1016/j.cjco.2025.09.009

**Published:** 2025-09-23

**Authors:** Thibaut Moulin, Nicolas Lellouche, Estelle Gandjbakhch, Mikael Laredo

**Affiliations:** a Université Paris-Est Créteil (UPEC), AP-HP Henri Mondor Hospital, Créteil, France; b Sorbonne Université, AP-HP, Pitié Salpêtrière Hospital, Paris, France

**Keywords:** Electrocardiogram, electrocardiogram teaching, medical education, microlearning, e-learning, digital learning

## Abstract

**Background:**

Electrocardiogram (ECG) interpretation is a critical skill for medical students that requires regular practice to achieve competency. Microlearning is an emerging pedagogical trend that offers students repetitive, short, and focused e-learning sessions. This study aimed to assess the feasibility of a 6-week, daily, digital ECG training program based on microlearning principles among undergraduate medical students.

**Methods:**

We conducted a bicentric noncontrolled pilot study. Volunteer medical students received a daily (from Monday to Friday) ECG quiz via commonly used digital platforms, followed by immediate feedback, for 6 weeks. The primary endpoint was the daily participation rate. Skill improvement was evaluated through a baseline test and a final test (20 questions, score ranging from 0-20). Student satisfaction and self-assessment of progression were measured.

**Results:**

A total of 47 students were included. The median daily participation rate was high, at 80.9% (iinterquartile range 73.9-86.2), but it tended to decrease over time (weeks 1-2, 87.2%; weeks 3-4, 81.2%; weeks 5-6, 70.2%). A comparison of baseline and final test scores showed a significant improvement, of 1.1 points (95% confidence interval 0.15-2.1; *P* = 0.03), after the program. No significant correlation was found between individual participation rate and score improvement. Overall, 93% of students subjectively perceived progression, and 93% were satisfied with the training program.

**Conclusions:**

Daily quiz-based microlearning is a feasible method to support ECG training, with high initial adherence. Future controlled studies are required to evaluate the impact of integrating this approach with traditional teaching methods and assess its long-term efficacy and sustainability.

## Central Illustration

**Figure:**
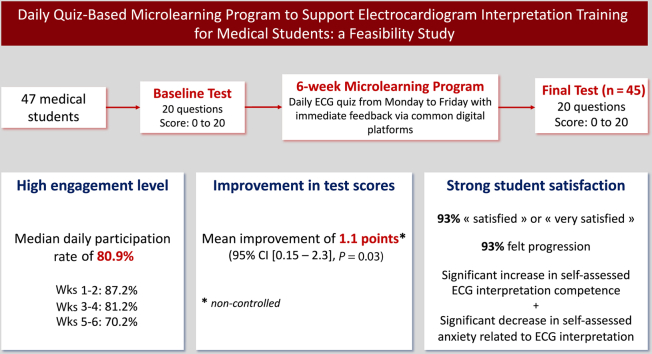

## Résumé

**Contexte:**

L'interprétation de l'électrocardiogramme (ECG) est une compétence essentielle pour les étudiants en médecine, qui nécessite une pratique régulière pour être maîtrisée. Le micro-apprentissage est une nouvelle tendance pédagogique qui propose aux étudiants des sessions d'apprentissage en ligne répétitives, courtes et ciblées.

Cette étude visait à évaluer la faisabilité d'un programme de formation numérique quotidien de 6 semaines sur les ECG, basé sur les principes du microapprentissage, auprès d'étudiants en médecine.

**Méthodologie:**

Nous avons mené une étude pilote bicentrique non contrôlée. Des étudiants en médecine volontaires ont reçu un test éclair quotidien sur l'ECG via des plateformes numériques couramment utilisées, suivi d'une rétroaction immédiate, pendant 6 semaines, du lundi au vendredi. Le critère d'évaluation principal était le taux de participation quotidien. L'amélioration des compétences a été évaluée à l'aide d'un test de référence et d'un test final (20 questions, score allant de 0 à 20). La satisfaction des étudiants et leur auto-évaluation de leurs progrès ont été mesurées.

**Résultats:**

Au total, 47 étudiants ont été inclus. Le taux de participation quotidien médian était élevé, à 80,9 % (écart interquartile 73,9-86,2), mais avait tendance à diminuer au fil du temps (semaines 1-2 : 87,2 %; semaines 3-4 : 81,2 %; semaines 5-6 : 70,2 %). En comparant les scores des tests initiaux et finaux, nous avons observé une amélioration significative de 1,1 point (intervalle de confiance à 95 % : 0,15-2,1; *p* = 0,03) à la fin du programme. Aucune corrélation significative n'a été trouvée entre le taux de participation individuel et l'amélioration des scores. Dans l'ensemble, 93 % des étudiants ont perçu subjectivement une progression et 93 % se sont déclarés satisfaits du programme de formation.

**Conclusions:**

Le microapprentissage quotidien basé sur des tests éclairs est une méthode viable pour soutenir la formation à l'ECG, permettant d'obtenir un taux d'adhésion initial élevé. Des études contrôlées supplémentaires sont nécessaires pour évaluer l'impact de l'intégration de cette approche aux méthodes d'enseignement traditionnelles et pour évaluer son efficacité et sa durabilité à long terme.

Twelve-lead electrocardiogram (ECG) interpretation is a complex yet essential skill in medical practice, crucial for diagnosing, risk-stratifying, and making decisions regarding potentially severe cardiac conditions. Given its wide accessibility and multiple applications, ECG recording and interpretation are no longer confined to the work of cardiologists, and all medical students are expected to achieve competency before graduating. However, students’ ability to interpret ECGs often falls short of expected proficiency,^1^^,^^2^ potentially leading to frustration^3^ and inappropriate clinical decisions.^4^

Although the didactic, lecture-based method remains predominant, no standard has ever been established for teaching ECG interpretation.^5^ In recent years, online teaching methods have been growing steadily among the digitally native generation of students (Generation Z or Millennial learners).^6^^,^^7^ E-learning solutions provide students with greater flexibility in terms of when, where, and how long they engage with the teaching material.^8^ Among these solutions, microlearning is an emerging pedagogical trend that enables students to engage in repetitive, short, focused, asynchronous, and mobile learning, which has shown promising results in medical education by helping to enhance engagement and knowledge retention.^9^^,^^10^ Conventionally, microlearning sessions last only a few minutes and can be accessible on-the-go by students via mobile platforms.^11^ ECG teaching seems well suited to microlearning; ECG tracings can be shared easily in image format, and interpreting a single ECG rarely takes more than a couple of minutes.

The reading of several hundred ECGs is recommended to become competent in ECG interpretation, and reading at least 100 ECGs per year is necessary to maintain this competency.^12^ Reaching this target requires some degree of self-directed learning from the students, which has been shown to have limitations in ECG interpretation teaching.^13^^,^^14^ A directed program of interpreting one ECG per day via a mobile platform could help students practice regularly and easily. However, the level of student adherence as well as information retention with such a program remain unknown. Data on the feasibility and efficacy of an ECG teaching method using digital repetitive microlearning are scarce. A single study showed that repetitive exposure of medical students to ECG reading significantly improved interpretation skills.^15^

We developed a 6-week program of online, daily, quiz-based ECG training for undergraduate medical students. The main objective of this pilot study was to assess the feasibility of the program through monitoring of student participation. The secondary objectives were to assess progression in ECG interpretation skills, report self-assessed outcomes, such as overall satisfaction and self-confidence in ECG interpretation, and identify specific areas of ECG interpretation associated with lower scores.

## Methods

### Educational intervention

We developed a daily, quiz-based ECG training program using microlearning principles. The requirements for the program were as follows: (i) easy setup and replication, without the need for a dedicated Web application; (ii) simplified mobile access via a Web link without a login password; and (iii) the ability to monitor daily participation.

Each morning at 8:00 AM, students received a link to a Google Forms survey (Google, Mountain View, CA) via a common messaging platform (WhatsApp, WhatsApp, Menlo Park, CA). The survey included a high-resolution ECG followed by a multiple-choice question (MCQ) with 5 response options, including multiple correct responses. Students were required to enter a unique personal anonymous identifier, enabling us to track their participation. After submitting their answer, students accessed a detailed, annotated response (approximately 10 to 20 lines), prepared by 2 experienced ECG educators, ensuring immediate feedback. Students also were asked to rate the difficulty of the question and the clarity of the response on a scale from 1-5. The link to the survey was accessible for 24 hours, without systematic reminders. The only systematic intervention from the administrators of the WhatsApp group was a motivational post each Friday, summarizing the weekly participation. Students no longer had access to the files after completing the survey, but they were free to take screenshots. The diagnoses and an example of the daily quizzes are available in Supplemental Table S1 and Supplemental Figure S1.

The daily ECG quiz program lasted 6 weeks, providing one ECG per day from Monday to Friday, totaling 30 ECGs. This format was designed to meet the expected competency goal in the curriculum for the students, focusing on key ECG interpretation skills necessary for medical practice.

### Study design

This single-arm, bi-centric study was conducted at Sorbonne Université, Paris, France and Paris-Est Créteil Université, Créteil, France. Volunteer undergraduate medical students engaged in the cardiology module were included. The protocol was approved by the ethics committees of both universities, and each participant provided written consent.

Prior to the start of the program, participants were given a unique identifier and completed an initial online test assessing their ECG interpretation skills, referred to here as the baseline test. This test included 20 ECG tracings with 14 MCQs and 6 short-answer questions. The scoring system for MCQs was as follows: 1 point for each fully correct answer; 0.5 points for answers with 1 incorrect choice; and 0.2 points for answers with 2 incorrect choices. Short-answer questions were scored as either 0 or 1 point. The individual score and test corrections were not given to the students.

The daily ECG quiz program started immediately after the baseline test and consisted of 30 quizzes, referred to here as Q1-Q30. Daily participation was defined as completing the daily ECG quiz, with monitoring based on each participant’s identifier, required to submit the form. A final test was administered 1 week after the 6-week program. This test followed the same format as the baseline test, featuring the same diagnoses and question items, but different ECG tracings. The range of the baseline and final tests was consequently 0-20.

Questions constituting the baseline and final tests were categorized as being relative to tachycardia, bradycardia, or repolarization analysis (Supplemental Table S2). Both baseline and final tests included the same amount of the 3 categories (tachycardia, 12; bradycardia, 5; repolarization analysis, 3).

Both the baseline and final tests included a self-evaluation of the anxiety related to ECG interpretation (rated on a scale of 1-10) and a self-assessment of ECG interpretation competence (rated on a scale of 1-10). Additionally, a satisfaction survey was conducted at the end of the program.

The primary endpoint was the daily participation rate. The secondary endpoints were as follows: progression, defined as the difference between the score obtained at the final test and the score obtained at the baseline test, for each student; scores obtained at the baseline and final tests according to each question’s category; and self-evaluated measurements that included satisfaction with the correction of each daily ECG quiz (rated on a scale of 1-5), perceived difficulty of each daily ECG quiz (rated on a scale of 1-5), self-assessment of ECG interpretation competence (rated on a scale of 1-10), anxiety related to ECG interpretation (rated on a scale of 1-10), overall satisfaction toward the educational program (categorized as very satisfied, satisfied, neutral, dissatisfied, and very dissatisfied), and time spent daily on learning.

### Statistical analyses

Continuous variables were expressed as median (interquartile range [IQR]) or mean ± standard deviation. Comparisons across groups were performed using the Kruskal-Wallis test;. The Steel-Dwass test was used to perform multiple one-to-one comparisons while accounting for alpha inflation risk. Paired comparisons of continuous variables were performed using the Wilcoxon matched-pairs signed rank test. Correlations between continuous variables were assessed using Spearman’s *r* coefficients. All statistical tests were 2-sided and were considered statistically significant at *P* < 0.05. Statistical analyses were performed with JMP, version 15.2.0 (SAS Institute, Cary, NC), and graphs were created with GraphPad PRISM, version 10.2.3 (Dotmatics, Boston, MA).

## Results

### Study population

In total, 47 medical school students in the Paris area (Sorbonne Université, 34; Paris-Est Créteil Université, 13) volunteered to participate and completed the baseline test. A total of 29 students were in year 3 (corresponding to year 2 of the North American MD program—ie, last preclinical year); 13 were in year 4 (corresponding to year 3 of the North American MD program—ie, core clerkship year); and 5 were in year 6 (corresponding to the first year of residency) of medical school.

### Participation

**Figure 1:**
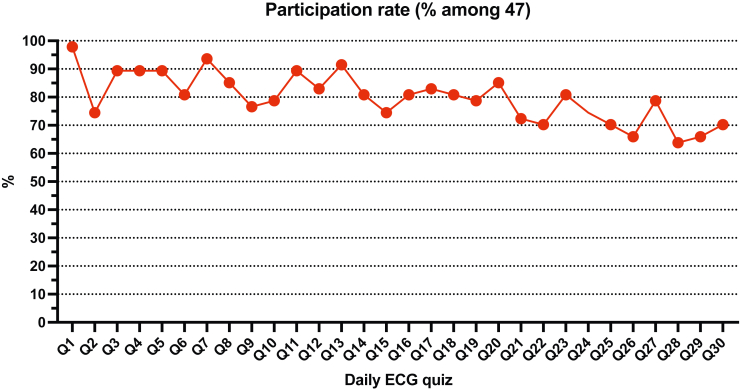
Participation in the daily electrocardiogram (ECG) quizzes (Qs). Daily participation rate calculated as % of the 47 students who volunteered to participate in the program and fulfilled the baseline test.

Daily participation is shown in Figure 1. The median daily participation rate was 80.9% (IQR 73.9-86.2). Participation significantly decreased during the 6-week daily ECG quiz period. When dividing the 6-week period into 2-week periods, the median participation rate was as follows: 87.2% (IQR 78.2%-90.4%) for week 1-2; 81.2% (IQR 80.3%-86.2%) for week 3-4; and 70.2% (IQR 66.0%-75.5%) for week 5-6 (*P* = 0.002 for week 5-6 vs week 3-4; *P* = 0.77 for week 3-4 vs week 1-2). No significant difference occurred in participation rate according to the year or university. A modest but significant correlation was found between individual participation rate and average daily score during the daily ECG quiz period (*r* = 0.42; *P* = 0.003).

### Daily ECG quizzes

On average, the median score for daily ECG quiz was 0.45 (IQR 0.36-0.55). Students in year 6 (0.66 [IQR 0.60-0.71]) performed significantly better than students in year 4 (0.43 [IQR 0.31-0.48], *P* = 0.0015) and students in year 3 (0.45 [IQR 0.37-0.54], *P* = 0.002), whereas no significant difference occurred between students in year 4 and students in year 3.

### Baseline and final tests

**Figure 2:**
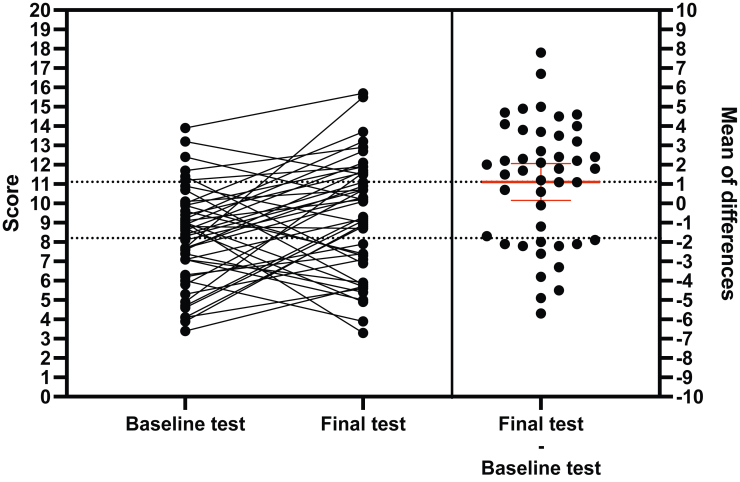
Assessment of progression in electrocardiogram interpretation. **Left panel**: matched scores from the baseline and final tests. **Right panel:** difference between scores on the final and baseline tests, for each student. The **thicker red line** is the mean of differences and the **whiskers** show the 95% confidence interval.

All 47 students completed the baseline test. Rated on a scale of 0-20 (1 point maximum per question), the median score was 8.4 (IQR 6.3-9.9; Fig. 2, left panel). Students in year 6 (12.4 [IQR 10.6-14.0]) performed significantly better than students in year 4 (7.1 [IQR 4.5-9.0], *P* = 0.01) and students in year 3 (8.4 [IQR 7.1-9.5], *P* = 0.003), whereas no significant difference occurred between students in year 4 and students in year 3.

A total of 45 students (96%) completed the final test. The median score was 9.3 (IQR 7.0-11.6; Fig. 2, left panel). Students in year 6 (11.9 [IQR 10.9-14.7]) performed significantly better than students in year 4 (7.1 [IQR 5.7-10.8], *P* = 0.04), whereas no significant difference occurred between students in year 4 and students in year 3, or between students in year 6 and students in year 3 (9.2 [IQR 7.4-11.6], *P* = 0.07). A modest correlation was found between scores on the baseline test and the final test (*r* = 0.36; *P* = 0.02).

### Progression

When comparing baseline and final test scores of the 45 students who completed both tests, a small but significant progression was found of 1.1 point on average (95% confidence interval [CI] 0.15-2.1; *P* = 0.03; Fig. 2, right panel). No significant difference occurred in progression according to the year in medical school (year 3, 1.17 points, 95% CI –0.1-2.5]; year 4, 1.22 points, 95% CI –0.8-3.2]; year 6, 0.46 points, 95% CI –2.6-3.5]).

**Figure 3:**
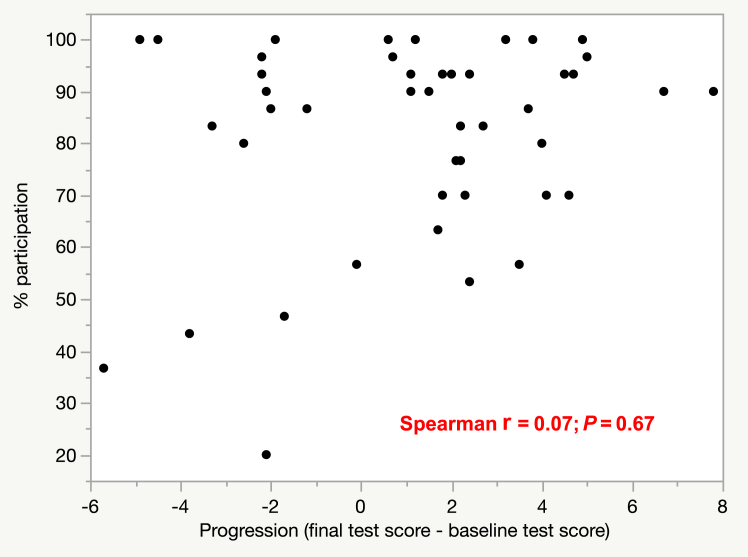
Correlation matrix between individual participation in daily electrocardiogram quizzes and score improvement.

No significant correlation occurred between individual participation rate and progression (*r* = 0.07; *P* = 0.67; Fig. 3), or with score on the baseline test (*r* = 0.21; *P* = 0.15), or score on the final test (*r* = 0.25; *P* = 0.10).

### Self-assessed outcomes

#### Daily ECG quizzes

The mean satisfaction level regarding the correction of daily ECG quizzes (rated on a scale of 1-5) was 4.38 ± 0.32 among 908 answers; the mean perceived difficulty level (rated on a scale of 1-5) was 3.32 ± 0.63 among 938 answers (Supplemental Fig. S2). A robust correlation was found between the perceived difficulty level and the score obtained daily (*r* = –0.85; *P* < 0.0001; Supplemental Fig. S2).

#### Overall program

Before the start of the program, the median self-assessed competence score in ECG interpretation (rated on a scale of 1-10) was 5 (IQR 4-6); this score was 6 (IQR 5-6.5) after completion of the program, corresponding to a significant increase of 0.82 points on average (95% CI 0.42-1.23; *P* < 0.0001). Before the start of the program, the anxiety level related to ECG interpretation (rated on a scale of 1-10) was 5 (IQR 3-7); whereas it was 3 (IQR 2-5) after completion of the program, corresponding to a significant decrease of 0.87 points on average (95% CI 0.17-1.57; *P* = 0.02).

A total of 29 students (64%) were very satisfied with the program overall; 13 (29%) were satisfied; and 3 (7%) expressed a neutral opinion. None expressed dissatisfaction. A total of 42 students (93%) felt that they progressed in ECG interpretation. A total of 41 students (91%) considered the daily ECG density to be “appropriate,”; 2 (4%) considered it to be “not enough”; and 2 (4%) considered it to be “too much.”

The time spent daily on the ECG quiz was 5.4 ± 2.6 minutes. The platform used was a smartphone for 42 students (93%), and a computer for the remaining 3 (7%). When asked what the 2 most common locations were in which daily ECG quizzes were undertaken, 29 students (64%) declared on public transportation, followed by a library (18; 40%), and the site of their ground hospital internship (21; 47%).

### Performance according to ECG category

When pooling the baseline and final tests, the score (0-20 range) obtained on tachycardia questions was significantly lower than the score obtained on bradycardia questions (7.6 [IQR 5.3-9.7] vs 10.7 [IQR 8.3-14.0], *P* < 0.0001).

## Discussion

In this non-controlled pilot study, we aimed to assess the feasibility of a novel educational intervention for ECG training, which involved a daily digital quiz over a 6-week period, based on microlearning principles. The main results are as follows (Central Illustration):

• The median daily participation rate was high, at 80.9%, ensuring the feasibility of such a program. However, participation decreased progressively throughout the 6-week period.
• We observed a small but significant improvement in ECG interpretation skills after the program, which did not seem to be associated with the participation level.
• A large majority of students subjectively declared that they improved in ECG interpretation, and students were vastly satisfied with the program.
• Medical students seemed to have more difficulty with tachycardia than with bradycardia reasoning.

**Central Illustration:**
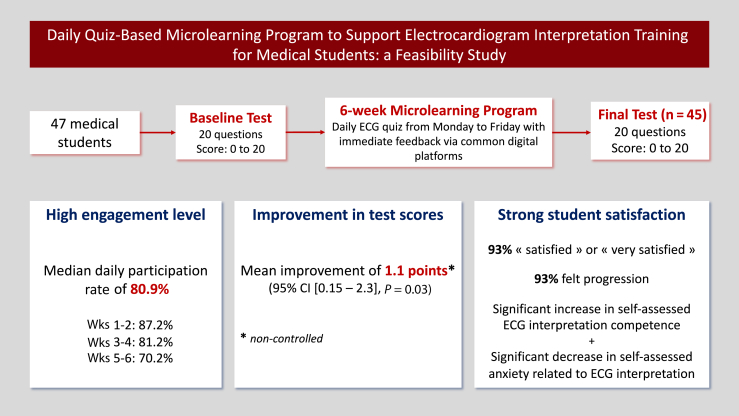
Daily quiz-based microlearning program to support electrocardiogram (ECG) interpretation training for medical students: a feasibility study. CI, confidence interval.

The high participation rate indicates that students are willing to engage with this type of learning method, suggesting strong initial adherence to the program. This approach appears to be compatible with students’ daily routines, allowing them to participate in short learning sessions that fit into their schedules. However, we noted a progressive drop in participation over time, consistent with findings from other educational interventions in which maintaining high engagement levels over time has proven to be a common challenge.^16^, ^17^, ^18^ To sustain participation, additional strategies may be needed, such as varying the content, incorporating regular reminders, or even using rewards.^19^ Finally, allowing students to access the quizzes for more than 24 hours would likely have increased the participation rate, but this approach could deviate from the concept of microlearning if students clustered their training sessions.

Microlearning refers to short and repetitive e-learning modules presented as text, images, videos, podcasts, or quizzes. One of its primary advantages is its asynchronous nature, enabling students to connect anytime and anywhere through universally accessible digital means.^11^ This flexibility enhances their engagement in learning and improves adherence to educational content. Few studies have evaluated microlearning in medical education to date. Consistent with our findings, all students reported high participation levels, regardless of the delivery method.^9^

Although statistically significant, the educational significance of the modest improvement in ECG interpretation skills that we observed is unclear. This result should be interpreted with caution, due to the noncomparative nature of the study. The relatively modest nature of the improvement may be due to the program's lack of integration into a comprehensive standard teaching curriculum, which includes essential lecture-based instruction, for understanding the complex pathophysiological concepts underlying ECG interpretation and enabling more effective practice and training.^20^^,^^21^ Indeed, other authors argue that the primary benefit of microlearning is to refresh existing skills that are used infrequently, rather than to acquire new skills.^9^ Nonetheless, the strong positive evaluation and perceived progression reported by students are promising indicators, even though several studies have shown that students' perception of progression does not always correlate with actual learning outcomes.^20^

Microlearning in medical education appears to enhance factual knowledge and learner satisfaction when it is delivered as short, focused modules or within mixed onsite and digital formats, the latter generally being reported as more effective than online-only approaches.^9^ Microlearning has been particularly studied in radiology, a discipline that shares with ECG interpretation the need for rapid visual analysis and structured reasoning, and measurable gains in learner performance have been shown in this field.^22^ In a randomized study, Hempel et al. reported that brief, case-based ultrasound screencasts yield significantly higher pretest scores (median 84.2 vs 65.8) than traditional lectures, but this difference did not translate into superior practical skills or knowledge retention.^23^ Vavasseur et al. showed that a mixed (classroom and short videos), self-paced approach improved posttest scores of abdominal imaging, with the greatest benefit seen in lower-performing students.^24^ In a comparative study by Belfi and colleagues, a “flipped blended-learning” approach using pre-class micro-e-learning modules significantly improved knowledge gains in medical imaging interpretation over lectures alone, was well received by students, and provided flexible, interactive learning.^25^

Longitudinal assessment has important implications for evaluating learning retention.^26^^,^^27^ In a recent study by Cunningham et al, medical students in a longitudinal integrated clerkship who completed periodic, spaced, online ECG quizzes throughout their clinical year achieved significantly higher posttest scores than those of peers receiving didactics alone, with large effect sizes for both control comparisons.^15^ The intervention, grounded in spaced repetition and retrieval practice, effectively maintained ECG interpretation skills and offers a model that could be adapted to reinforce other clinical reasoning competencies. This study illustrates how sustained competency in this domain requires reinforcement over time, to counteract the forgetting curve. Future studies evaluating ECG microlearning on a broader scale should incorporate a longitudinal evaluation to assess the retention of interpretation skills.

The findings of our study have important practical implications for ECG teaching. Integrating daily microlearning quizzes into the standard curriculum could provide a consistent and engaging method to support students’ training in ECG interpretation skills. Considering that reading several hundred ECGs is recommended to achieve competence,^12^ this method offers a practical means, among others, for students to practice regularly, although the extent of long-term participation needed to reach these numbers remains uncertain. The program's ease of implementation and replication, facilitated by commonly used digital platforms, underscores its practical utility. Finally, the detailed, annotated responses provided after each quiz likely contributed to the students' learning and satisfaction, offering immediate feedback, which has been shown to be essential for information retention in e-learning approaches.^20^

### Limitations

This study has several limitations. First and foremost, the absence of a control group limits our ability to directly attribute the observed improvements to the microlearning program alone. A limitation in comparing the baseline and final tests was the lack of difficulty calibration between them. Each included 20 questions, a number too small to limit variability, yet a more comprehensive test risked losing students’ attention in this optional program and yielding results that might not reflect their interpretation skills accurately. Second, students were simultaneously engaged in other activities, including clinical rotations and concurrent lectures, which varied by year and between the 2 universities. These differences may have acted as confounders in the modest improvement observed in interpretation skill scores. Third, as mentioned above, no longitudinal assessment was made of mid- and long-term learning retention, which is of major importance when evaluating time-dependent educational interventions. Fourth, we found no significant correlation between participation rates and either final test scores or score improvement, suggesting that factors other than participation alone, aside from a possible lack of statistical power, may influence learning outcomes. The study's 6-week duration also limits our understanding of the long-term effects and sustainability of this educational approach. Fifth, the relatively small sample size and lack of demographic diversity limit the generalizability of the findings. Last, the reliance on voluntary participation and the use of self-reported satisfaction and progression data pose the risk of inherent biases.

## Conclusions

Daily quiz-based microlearning is a feasible method to enhance ECG interpretation training, achieving a high level of initial participation and significant satisfaction among undergraduate medical students. However, maintaining long-term engagement remains a challenge. The modest short-term improvement in ECG interpretation skills highlights the need for integrating microlearning training with comprehensive traditional teaching methods. Future controlled studies, ideally randomized, are required to investigate the impact of such blended approaches and assess their long-term benefits and sustainability.
