# Supplementary material for: Daily Quiz-Based Microlearning Program to Support Electrocardiogram Interpretation Training for Medical Students: A Feasibility Study
Source: CJC Open. 2025 Sep 23;8(1):43–50. doi: 10.1016/j.cjco.2025.09.009 (PMC12925806; doi:10.1016/j.cjco.2025.09.009)
Supplement: Supplementary Tables 1 and 2 and Supplementary Figures 1 and 2 [file mmc1.docx]

**Supplemental Material**

**Supplemental Table S1: Diagnoses of daily ECG quizzes.**

| **Quiz** | **ECG diagnosis** |
| --- | --- |
| Q1 | RBBB and left fascicular posterior block |
| Q2 | SVT |
| Q3 | LBBB |
| Q4 | Atrial premature beat |
| Q5 | Slow AF and LBBB |
| Q6 | 2:1 AV block |
| Q7 | Atrial flutter |
| Q8 | Third-degree AV block |
| Q9 | Atrial tachycardia and effect of vagal maneuvers |
| Q10 | Mobitz type I second-degree AV block |
| Q11 | Rapid AF and LBBB |
| Q12 | Third-degree AV block and inferior STEMI |
| Q13 | VT |
| Q14 | Ventricular pre-excitation |
| Q15 | Atrial Flutter |
| Q16 | Sino-atrial block |
| Q17 | SVT |
| Q18 | AF |
| Q19 | AF and third-degree AV block |
| Q20 | Sinus tachycardia |
| Q21 | Sino-atrial block |
| Q22 | Acute pericarditis |
| Q23 | Left ventricular hypertrophy |
| Q24 | AF and ventricular pre-excitation |
| Q25 | Hyperkaliemia |
| Q26 | SVT and effect of adenosine injection |
| Q27 | Mobitz type I second-degree AV block |
| Q28 | Atrial premature beat |
| Q29 | Third-degree AV block |
| Q30 | SVT and effect of adenosine injection |

RBBB: right bundle branche block; SVT: supraventricular tachycardia; LBBB: left bundle branche block; AF: atrial fibrillation; AV: atrioventriculaire; STEMI: ST-elevation myocardial infarction; VT: ventricular tachycardia.

**Supplemental Table S2: Diagnoses of baseline and final tests**

| **Category** | **ECG diagnosis** |
| --- | --- |
| « Tachycardia » | AF and left ventricular hypertrophy |
|  | AF and LBBB |
|  | AF and ventricular pre-excitation |
|  | Atrial flutter |
|  | Atrial premature beat |
|  | SVT |
|  | SVT |
|  | SVT and effect of adenosine injection |
|  | Sinus tachycardia |
|  | VT |
|  | VT |
|  | Ventricular pre-excitation |
| « Bradycardia » | Third-degree AV block |
|  | Mobitz type I second-degree AV block |
|  | Sino-atrial block |
|  | AF and third-degree AV block |
|  | RBBB and left fascicular block |
| « Repolarisation » | Territory of T-wave inversion |
|  | STEMI |
|  | Acute pericarditis |

RBBB: right bundle branche block; SVT: supraventricular tachycardia; LBBB: left bundle branche block; AF: atrial fibrillation; AV: atrioventriculaire; STEMI: ST-elevation myocardial infarction; VT: ventricular tachycardia.

**
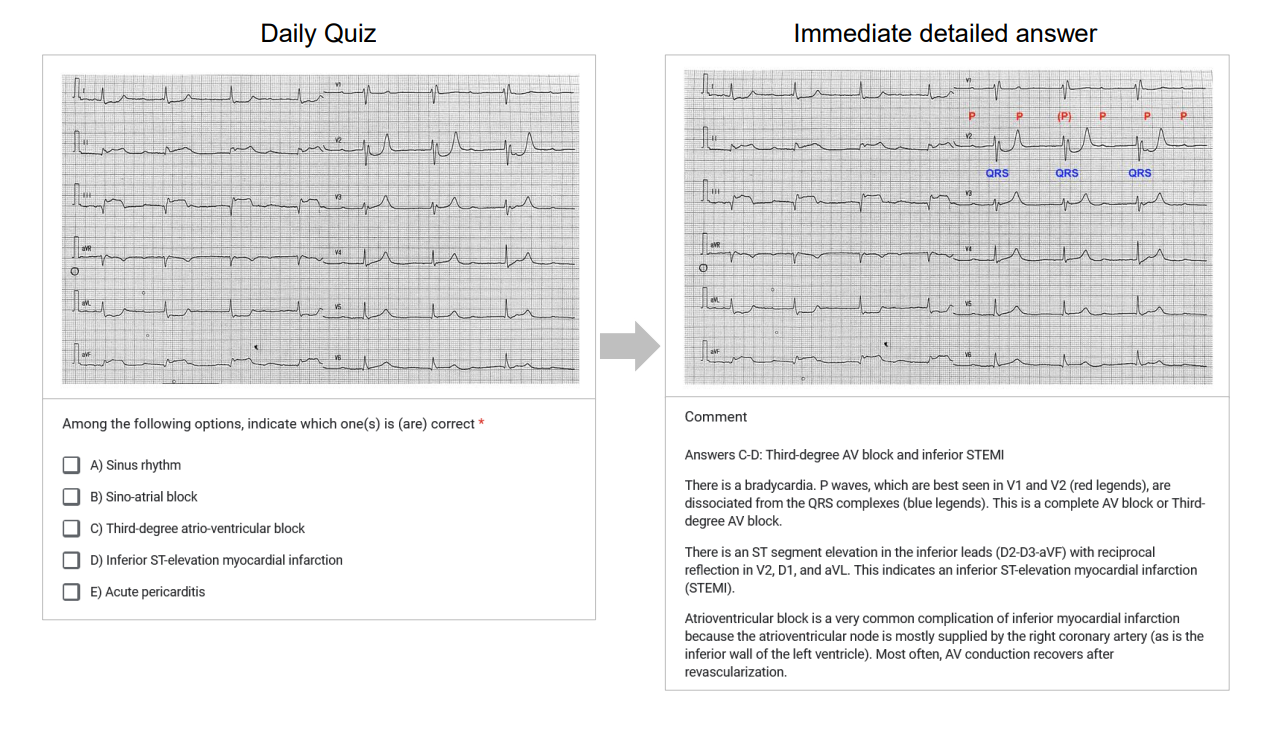
**

**Supplemental Figure S1: Exemple of daily ECG quiz (translated from French to English).**

**
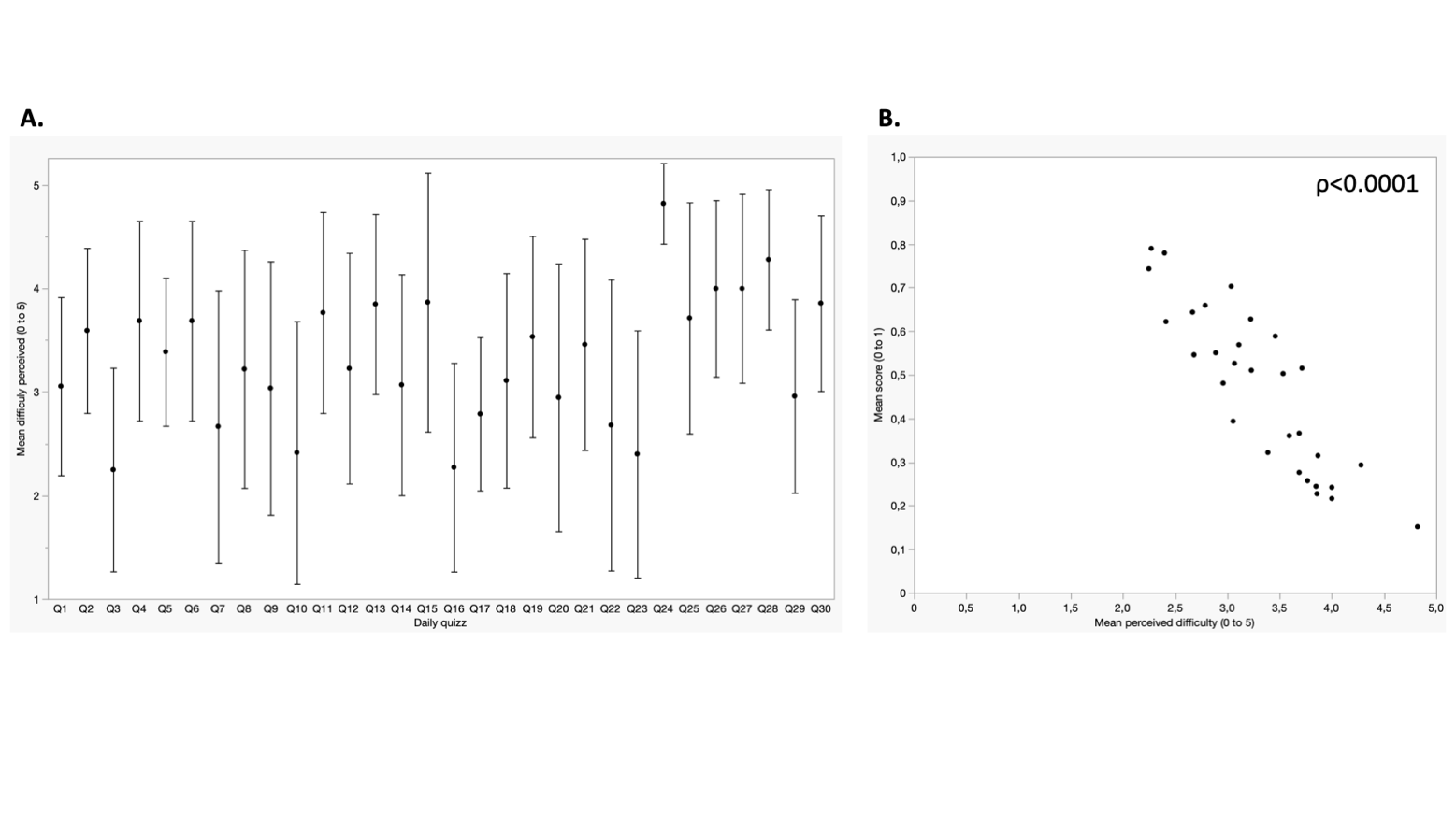
** **Supplemental Figure S2: Subjective difficulty assessment and its relation to mean score.**

**A.** Mean daily difficulty perceived subjectively graded from 0 to 5 for each daily quizz. The dot refers to the mean and the bar to the standard deviation. The overall mean difficulty was 3.32±0.63 on 938 observations. **B.** Correlation between mean score and mean perceived difficulty for each daily quizz. The spearman correlation coefficiant was <0.0001.
